# Editorial: Compulsory Interventions in Psychiatry: An Overview on the Current Situation and Recommendations for Prevention and Adequate Use

**DOI:** 10.3389/fpsyt.2020.622373

**Published:** 2020-12-07

**Authors:** Christian G. Huber, Andres R. Schneeberger

**Affiliations:** ^1^Universitäre Psychiatrische Kliniken (UPK) Basel, Klinik für Erwachsene, University of Basel, Basel, Switzerland; ^2^Psychiatric Services Grisons, Chur, Switzerland; ^3^Department of Psychiatry, Psychotherapy and Psychosomatics, Psychiatric Hospital, University of Zurich, Zurich, Switzerland; ^4^Department of Psychiatry and Behavioral Sciences, Albert Einstein College of Medicine, New York, NY, United States

**Keywords:** coercion, autonomy, involuntary treatment, seclusion, restraint, involuntary admission, community treatment order

State-of-the-art clinical psychiatry seeks to provide successful treatment of persons with mental illness in a comprehensive approach integrating biological, psychological, social, and spiritual aspects (1). The focus on empowerment- and recovery-oriented strategies allows clinicians and people with mental illness to interact at the same level (2). However, illness symptoms sometimes prevent patients from fully understanding the potential benefits of treatment (3, 4). Aggression, violence, and self-endangering behavior in psychiatric patients are often used as justification for more restrictive policies in mental health care (4–7). Yet, current studies have shown that restrictive settings do not necessarily prevent self-harm and suicide (8, 9) and do not reduce all sorts of violence and aggression (10). Locked doors lead to a worse climate on the wards, directly affecting the therapeutic milieu and the treatment alliances (11, 12). In addition, patients who were mandated to psychiatric treatment might be more reluctant to receive treatment in the future [(3); Hachtel et al.]. While studies have shown that mandated treatments do also positively impact the outcome of illness, this is often mediated by an increase in services rendered rather than the direct effects of coercion (13).

Compulsory interventions aiming at patient and staff safety, as well as mandatory treatment may be necessary to ensure treatment for those who do not want to be treated (4). The goal is to protect mentally ill persons from self-harm, suicide, and detrimental consequences of untreated illness, and to protect relatives, healthcare professionals, and the general public from preventable aggression and violence (14). This gives rise to serious ethical problems and clinical challenges.

In the current Research Topic, Hoff elaborates on these ethical challenges. He discusses that coercive interventions have to be considered exceptional measures and may only be used under well-defined ethical and juridical conditions. Although guidelines should be adhered to, they cannot substitute individual case-based decisions. Furthermore, he recommends taking on the debate on autonomy in psychiatry, as facing this challenge might prove beneficial for psychiatry and its patients. Also focusing on patient autonomy, Scholten et al. discuss how different interpretations of psychiatric advance directives (PAD) may be useful for patients in future crises. They critically discuss interpretations of PAD promoting and undermining autonomy and propose that using supported decision-making and competence assessment may help to employ PAD successfully. As—like Hoff states—guidelines can only give general recommendations, Montaguti et al. discuss how clinical ethics consultation can be helpful to inform case-based decisions on coercive measures.

Following up to these ethical considerations, Oliva et al. discuss the legal preconditions for coercive measures. They explore if the judicial basis and the actual reasons for compulsory admission to psychiatric treatment in Turin (Italy) are mutually compatible and the authors critically discuss if changes in legislation are necessary. Becker and Forman bring up the point that psychiatric emergencies often occur in emergency rooms and outside of specific psychiatric settings. They discuss the role of implied consent in these emergency settings and explore the legal and ethical basis for acting in emergencies. Finally, Hachtel et al. discuss how the legal basis of mandated treatment influences psychiatric therapy and treatment outcome. They distinguish between the concepts of formal vs. perceived coercion and examine how psychiatry could fulfill a dual mandate of control and therapy and how it can be helpful even in the context of formal coercion. McMillan et al. also explore the issue of building a positive therapeutic alliance in the context of coercion. Using qualitative analysis of interviews with persons subjected to community treatment orders (CTO) and mental health professionals, they debate the role of trust and mistrust for the success and failure of reaching recovery in the frame of a CTO.

The legal framework regulating compulsory interventions has a direct impact on psychiatric practice. Radisic and Kolla focus on the situation in Ontario (Canada), where psychiatric inpatients who appeal to the Consent and Capacity Board (CCB) have the right to refuse medication until the CCB has come to a decision. They examine the frequency of seclusion and restraint in civil and forensic inpatients during this time and discuss how improving legislation and time to CCB decision could be beneficial for these patients. Mahler et al. take a look at the interplay of legal procedures and structural factors in their influence on the use of coercive measures in Germany. Arnold et al. examine the situation in Basel (Switzerland) and evaluate factors associated with compulsory admissions to psychiatric wards and with appeals against these admissions. Finally, Buadze et al. explore the position of defense lawyers in Switzerland on the availability of opioid agonist treatment vs. forced discontinuation in pre-trial detention and prisons. In these settings, availability of a specific treatment is partly controlled and moderated by custodians and legal professionals. Thus, there is a risk that this patient population does not receive interventions according to psychiatric state-of-the-art guidelines.

Addressing issues from social psychiatry and mental health services research, the original papers in the current Research Topic employ a broad set of qualitative and quantitative methods. Data for many of the publications in this Research Topic stem from clinical routine documentation. In this context, Fröhlich et al. examine the reliability of paper-based routine documentation in psychiatric inpatient care and find acceptable reliability, depending on the chosen documentation categories and variables. Together with the published literature, this indicates that both electronic and paper-based routine documentation can be used for health services research, but that their limitations have to be kept in mind.

Several publications focus on the frequency of coercive measures. In an effort to improve an overview on the use and effects of compulsory measures in psychiatry, de Bruijn et al. present a protocol for a systematic review on physical and pharmacological restraints. Saya et al. provide a narrative review on criteria, procedures, and future prospects of involuntary treatment in psychiatry around the world. Legal and ethical views and mental health services structures and traditions vary depending on country and sometimes region where patients are treated—and they have a considerable impact on practices regarding compulsory interventions. Thus, information on the situation in different countries provides important information and the basis for a discussion how the different settings should be developed.

Concerning the effects of coercive measures, Chieze et al. present a systematic review on the effects of seclusion and restraint in adult psychiatry. They conclude that, although the heterogeneity of the included studies limits interpretation, the overall results show negative effects of seclusion and restraint, and that more research is needed. Kersting et al. summarize the current literature on physical harm and death in the context of coercive measures in psychiatry in a systematic review, taking a look at a highly relevant but currently underresearched aspect of coercion in psychiatry.

As compulsory measures can often be avoided if a critical situation can be identified and successfully addressed early enough, research on the prediction of coercion is highly needed. To enhance our knowledge on the predictors of compulsory admission to treatment, Marty et al. study the characteristics of psychiatric emergency situations and the decision-making process leading to involuntary admission, and Lay et al. analyze the predictors of compulsory re-admission to psychiatric inpatient care. Günther et al. use machine learning to identify direct coercion in a high-risk subgroup of forensic patients with schizophrenia. And Hazewinkel et al. and Stepanow et al. explore the possibility of predicting seclusion by analyzing text entries from routine documentation in electronic medical records.

Several papers in the current Research Topic examine approaches to prevent or reduce compulsory interventions in psychiatry. Zinkler and Brophy et al. discuss the possibility to use supported decision making in the prevention of compulsory interventions and of community treatment orders in mental health care. Baumgardt et al. (a) study the effects of the introduction of the Safewards model on the use of coercive measures on two locked wards in Germany [please note that this paper has been updated according to the corrigendum in Baumgardt et al. (b)]. Schöttle et al. report that the introduction of an integrated care model in Hamburg (Germany) is connected with a reduction of involuntary admissions in patients with severe psychotic disorders. As Rabenschlag et al. discuss from a nursing perspective, de-escalation strategies can be useful for preventing and reducing coercion in psychiatry—including informal coercion, which is otherwise often overlooked. The authors highlight the importance of the attitudes and values of the person perceiving aggression for their response to this behavior and advise that health care personnel should develop a critical awareness toward the use of coercive measures. Finally, Widmayer et al. pose the question if animal-assisted therapy could help to reduce coercive treatment in psychiatry and, based on positive findings in the current literature supporting this possibility, encourage future research on this topic.

Lastly, a number of papers assess staff and consumer perspectives on compulsory interventions in psychiatry. Sampogna et al. examine perceived coercion among inpatients of five psychiatric wards in Italy and its associations with treatment satisfaction. Efkemann et al. analyze ward atmosphere and patient satisfaction in locked, facultative locked and open door settings. Fletcher, Hamilton et al. explore the impact of the introduction of the Safewards model in inpatient mental health units in Victoria (Australia) on healthcare professionals and assess consumer perspectives in their second paper (Fletcher, Buchanan-Hagen et al.). Franke et al. address forensic psychiatric inpatient settings and examine perceived institutional restraint and psychological distress. Focusing specifically at substance use disorder wards, Steinauer et al. report healthcare personnel and consumer perspectives on the benefits and drawbacks of introducing an open door policy.

Concerning specific interventions and populations, Reisch et al. examine attitudes to containment measures of patients, next of kin, and health care professionals. Jaeger et al. conducted a qualitative study assessing the opinions of inpatients, their relatives, and healthcare professionals on not performing compulsory medication during involuntary inpatient treatment. Lastly, Soares and Pinto da Costa examined the experiences and perceptions of police officers concerning their interactions with people with serious mental disorders for compulsory treatment.

Table 1 gives an overview on the papers published within the scope of the current Research Topic.

**Table 1:** Overview on the papers published within the scope of the Research Topic.

**Topic/Authors**	**Title**	**Article type**
**Legal and ethical aspects**		
Hoff	Compulsory interventions are challenging the identity of psychiatry	Perspective
Scholten et al.	Psychiatric advance directives under the convention on the rights of persons with disabilities: why advance instructions should be able to override current preferences	Policy and Practice Reviews
Montaguti et al.	Reflecting on the reasons pros and cons coercive measures for patients in psychiatric and somatic care: the role of clinical ethics consultation. A pilot study	Original Research
Oliva et al.	Compulsory psychiatric admissions in an italian urban setting: are they actually compliant to the need for treatment criteria or arranged for dangerous not clinical condition?	Original Research
Becker and Forman	Implied consent in treating psychiatric emergencies	Opinion
Hachtel et al.	Mandated treatment and its impact on therapeutic process and outcome factors	Review
McMillan et al.	Trust and community treatment orders	Original Research
Radisic and Kolla	Right to appeal, non-treatment, and violence among forensic and civil inpatients awaiting incapacity appeal decisions in Ontario	Original Research
Arnold et al.	Compulsory admission to psychiatric wards–who is admitted, and who appeals against admission?	Original Research
Mahler et al.	Same, same but different: how the interplay of legal procedures and structural factors can influence the use of coercion	Opinion
Buadze et al.	The accessibility of opioid agonist treatment and its forced discontinuation in swiss prisons—attitudes, perceptions and experiences of defense lawyers in dealing with detained persons using opioids	Original Research
**Use of routine data**		
Fröhlich et al.	Reliability of paper-based routine documentation in psychiatric inpatient care and recommendations for further improvement	Original Research
**Frequency of coercive measures**		
de Bruijn et al.	Physical and pharmacological restraints in hospital care: protocol for a systematic review	Clinical Study Protocol
Saya et al.	Criteria, procedures, and future prospects of involuntary treatment in psychiatry around the world: a narrative review	Review
**Effects of coercion**		
Chieze et al.	Effects of seclusion and restraint in adult psychiatry: a systematic review	Systematic Review
Kersting et al.	Physical harm and death in the context of coercive measures in psychiatric patients: a systematic review	Systematic Review
**Prediction of coercion**		
Marty et al.	Characteristics of psychiatric emergency situations and the decision-making process leading to involuntary admission	Original Research
Lay et al.	Predictors of compulsory re-admission to psychiatric inpatient care	Original Research
Günther et al.	Identifying direct coercion in a high risk subgroup of offender patients with schizophrenia via machine learning algorithms	Original Research
Hazewinkel et al.	Text analysis of electronic medical records to predict seclusion in psychiatric wards: proof of concept	Original Research
Stepanow et al.	Narrative case notes have the potential to predict seclusion 3 days in advance: a mixed-method analysis	Original Research
**Prevention and reduction of coercion**		
Zinkler	Supported decision making in the prevention of compulsory interventions in mental health care	Opinion
Brophy et al.	Community treatment orders and supported decision-making	Original Research
Baumgardt et al. (a)	Preventing and reducing coercive measures—an evaluation of the implementation of the safewards model in two locked wards in Germany	Original Research
Schöttle et al.	Reduction of involuntary admissions in patients with severe psychotic disorders treated in the ACCESS integrated care model including therapeutic assertive community treatment	Original Research
Rabenschlag et al.	Nursing perspectives: reflecting history and informal coercion in de-escalation strategies	Perspective
Widmayer et al.	Could animal-assisted therapy help to reduce coercive treatment in psychiatry?	Mini Review
**Staff and consumer perspectives**		
Sampogna et al.	Perceived coercion among patients admitted in psychiatric wards: Italian results of the EUNOMIA study	Original Research
Efkemann et al.	Ward atmosphere and patient satisfaction in psychiatric hospitals with different ward settings and door policies. results from a mixed methods study	Original Research
Fletcher, Hamilton et al.	Safewards impact in inpatient mental health units in Victoria, Australia: staff perspectives	Original Research
Fletcher, Buchanan-Hagen et al.	Consumer perspectives of safewards impact in acute inpatient mental health wards in Victoria, Australia	Original Research
Franke et al.	Perceived institutional restraint is associated with psychological distress in forensic psychiatric inpatients	Original Research
Steinauer et al.	Opening the doors of a substance use disorder ward—benefits and challenges from a consumer perspective	Perspective
Reisch et al.	Comparing attitudes to containment measures of patients, health care professionals and next of kin	Original Research
Jaeger et al.	Refusing medication therapy in involuntary inpatient treatment—a multiperspective qualitative study	Original Research
Soares and Pinto da Costa	Experiences and perceptions of police officers concerning their interactions with people with serious mental disorders for compulsory treatment	Original Research

The primary focus of this Research Topic was to provide an overview on the current situation in clinical psychiatry and in psychiatric research, to collect scientific evidence on the prevention and adequate use of compulsory interventions, its effects and consequences. Now when finished, it also gives recommendations for mental health care professionals on the prevention of aggression and violence, the use of coercive measures and possible treatment alternatives to reduce forced interventions. In addition, it outlines future research strategies to advance the field and to ultimately approach the goal of optimal and safe treatment of this vulnerable population.

Of course, the number and distribution of submissions to a Research Topic cannot be considered representative for a research field. However, the considerable number of papers together with the current scientific literature show that research on compulsory interventions is a broad and active field in psychiatry. This mirrors a rising awareness of this issue in academic and clinical psychiatry. Ethical and legal aspects of coercion, the prediction, prevention and reduction of coercion, and consumer perspectives on coercion where the most prominent focus of submissions to this Research Topic. This shows that professionals within the field of psychiatry are critically evaluating in which situations and under which preconditions compulsory measures should be used and how they can be avoided. It acknowledges the importance of the field for healthcare professionals, patients, their relatives and the population, and shows that there is a willingness to strife for a minimal restrictive environment for the patients. From our view, this is a very positive development and fosters hope that we can successfully improve the situation for our patients and psychiatry now and in the future.

## Author Contributions

CH wrote the first draft of the manuscript. AS critically revised the manuscript and provided important intellectual contributions. All authors have read and approved the final version.

## Conflict of Interest

The authors declare that the research was conducted in the absence of any commercial or financial relationships that could be construed as a potential conflict of interest.
